# Automatic signal quality assessment of raw trans-abdominal biopotential recordings for non-invasive fetal electrocardiography

**DOI:** 10.3389/fbioe.2023.1059119

**Published:** 2023-02-27

**Authors:** Giulia Baldazzi, Eleonora Sulas, Rik Vullings, Monica Urru, Roberto Tumbarello, Luigi Raffo, Danilo Pani

**Affiliations:** ^1^ Department of Electrical and Electronic Engineering, University of Cagliari, Cagliari, Italy; ^2^ Department of Electrical Engineering, Eindhoven University of Technology, Eindhoven, Netherlands; ^3^ Pediatric Cardiology and Congenital Heart Disease Unit, ARNAS G. Brotzu Hospital, Cagliari, Italy

**Keywords:** signal quality assessment, fetal electrocardiography, ECG, non-invasive fetal ECG, fetal monitoring, machine learning

## Abstract

**Introduction:** Wearable monitoring systems for non-invasive multi-channel fetal electrocardiography (fECG) can support fetal surveillance and diagnosis during pregnancy, thus enabling prompt treatment. In these embedded systems, power saving is the key to long-term monitoring. In this regard, the computational burden of signal processing methods implemented for the fECG extraction from the multi-channel trans-abdominal recordings plays a non-negligible role. In this work, a supervised machine-learning approach for the automatic selection of the most informative raw abdominal recordings in terms of fECG content, i.e., those potentially leading to good-quality, non-invasive fECG signals from a low number of channels, is presented and evaluated.

**Methods:** For this purpose, several signal quality indexes from the scientific literature were adopted as features to train an ensemble tree classifier, which was asked to perform a binary classification between informative and non-informative abdominal channels. To reduce the dimensionality of the classification problem, and to improve the performance, a feature selection approach was also implemented for the identification of a subset of optimal features. 10336 5-s long signal segments derived from a real dataset of multi-channel trans-abdominal recordings acquired from 55 voluntary pregnant women between the 21st and the 27th week of gestation, with healthy fetuses, were adopted to train and test the classification approach in a stratified 10-time 10-fold cross-validation scheme. Abdominal recordings were firstly pre-processed and then labeled as informative or non-informative, according to the signal-to-noise ratio exhibited by the extracted fECG, thus producing a balanced dataset of bad and good quality abdominal channels.

**Results and Discussion:** Classification performance revealed an accuracy above 86%, and more than 88% of those channels labeled as informative were correctly identified. Furthermore, by applying the proposed method to 50 annotated 24-channel recordings from the NInFEA dataset, a significant improvement was observed in fetal QRS detection when only the channels selected by the proposed approach were considered, compared with the use of all the available channels. As such, our findings support the hypothesis that performing a channel selection by looking directly at the raw abdominal signals, regardless of the fetal presentation, can produce a reliable measurement of fetal heart rate with a lower computational burden.

## 1 Introduction

Portable and wearable solutions for continuously monitoring vital signs during daily-life activities allow unobtrusive data measurement outside the hospital. In such a context, advanced signal processing algorithms are required to provide reliable information to detect pathologic conditions (Majumder et al., 2017). This would allow monitoring both chronic patients and healthy people to prevent possible diseases (Gaikwad and Warren, 2009). Consequently, real-time telemonitoring of clinical parameters for immediate processing and early detection of symptoms is a hot research topic (Lanzola et al., 2014). Specifically, the adoption of wearable fetal monitoring systems by non-invasive multi-channel fetal electrocardiography (fECG) could be exploited during pregnancy, thus allowing early diagnosis and scheduling of *in-utero* treatment or postnatal intervention. In the last decades, some fetal heart rate (fHR) monitoring devices based on non-invasive fECG have been introduced (Behar et al., 2019; Kahankova et al., 2020; Rooijakkers and Springer, 2020). The first commercially available system has been the wireless Monica AN24 monitor by Monica Healthcare (Nottingham, United Kingdom), adopting conventional electrodes placed on the maternal abdomen. Then, disposable-patch systems were introduced, as the Novii Wireless Patch System by GE Healthcare (Chicago, Illinois, United States), the MERIDIAN M110 system by MindChild Medical (North Andover, Massachusetts, United States), the PUREtrace and the Nemo Fetal Monitoring System by Nemo Healthcare (Veldhoven, Netherlands). Moreover, in order to guarantee long-term continuous assessment of fetal wellbeing, many at-home monitoring technologies have been proposed as the wearable 5-channel monitor system by Bloomlife (San Francisco, California, United States and Genk, Belgium) and Imec (Leuven, Belgium and Eindhoven, Netherlands) featuring the first integrated circuit produced for mobile fECG monitoring, the FDA-cleared Invu device (Mhajna et al., 2020) by Nuvo (Tel Aviv, Israel), and the Owlet Band (Lehi, UT, United States). Generally, in these embedded systems, the power profile plays a key role to save battery and allow for longer monitoring time. In this sense, an important aspect is related to the signal processing method implemented for the extraction of the fECG signals from raw multi-channel trans-abdominal recordings. Indeed, several issues affect the non-invasive recording of fECG signals, as their low signal-to-noise ratio (SNR) (Peters et al., 2001; Sameni and Clifford, 2010; Clifford et al., 2014; Donofrio et al., 2014) due to the source and the propagation issues related to the fetal cardiac electrical activity, but also to different bioelectrical interferences (Jagannath and Selvakumar, 2014; Agostinelli et al., 2015) from the mother, particularly the maternal ECG (mECG). Noises and interferences overlap with the weaker fECG in various domains (Sameni and Clifford, 2010; Agostinelli et al., 2015), and especially in both time and frequency domains, thus requiring powerful signal processing methods for the fECG to be effectively recovered (Jaros et al., 2018).

The assessment of the raw input signal quality, to select the most informative channels for the subsequent processing, could represent a key step. Indeed, signal quality assessment (SQA) of non-invasive abdominal recordings could allow preserving only those channels exhibiting an adequate fECG content. In the past, SQA has been widely exploited on adult ECGs in order to reject those signals suffering from unacceptable noise level, and as such possibly leading to incorrect clinical interpretations (Del Rio et al., 2011; Satija et al., 2018). Different signal quality indexes (SQIs) were proposed and adopted, to allow for automatic accurate estimation of R peak (Johnson et al., 2015) and robust HR estimation (Li et al., 2007; Orphanidou et al., 2015), to reduce alarms associated to false arrhythmia and HR (Allen and Murray, 1996; Wang, 2002; Li and Clifford, 2012; Behar et al., 2013; Daluwatte et al., 2016; Shahriari et al., 2018), or, more generally, to identify clinically acceptable ECGs (Behar et al., 2012; Clifford et al., 2012; Di Marco et al., 2012; Zhao and Zhang, 2018), even in real-time monitoring mobile devices (Redmond et al., 2008; Langley et al., 2011; Moody, 2011; Silva et al., 2011; Hayn et al., 2012; Martinez-Tabares et al., 2012; Liu et al., 2018), or along with their noise level quantification (Johannesen and Galeotti, 2012; Li et al., 2014).

Nonetheless, besides discarding those “confounding” channels extremely affected by noise, the SQA could be helpful in reducing the computational burden of the fECG extraction algorithms, by limiting the number of channels to be processed. Obviously, this has consequences also on the architectural features for the processing core in charge to execute these algorithms, which could represent a hard specification for low-power portable fetal monitors, advocating the adoption of advanced signal processing platforms for pursuing real-time (Pani et al., 2013). Furthermore, both the time-varying fetal orientation and their movements make some channels useless in the fECG extraction process. As such, SQA has been used on fECG signals after their extraction to improve fHR estimation by signal quality metrics and artificial intelligence tools (Andreotti et al., 2017; Varanini et al., 2017; Fotiadou et al., 2021; Shi et al., 2022), but also to identify useful independent components after blind source separation algorithms for optimal fECG signal recovery (Karimi Rahmati et al., 2017; Jamshidian-Tehrani and Sameni, 2018). Nonetheless, SQA applied on the extracted fECG is biased by the effectiveness of the algorithms adopted for fECG extraction or fetal QRS detection (Mertes et al., 2022; Shi et al., 2022), which the SQI identification was based on. Indeed, some authors explored the adoption of SQA on the raw abdominal ECGs. Specifically, in (Liu et al., 2014) a single SQI was adopted to guarantee an accurate fetal and maternal QRS complexes location by considering a data-driven threshold before fECG extraction and QRS detection. Conversely, in (Mertes et al., 2022) the authors exploited the time-frequency representation of the abdominal signals to predict the quality of non-invasive fECG signals by deep convolutional neural networks (CNN), which however require power-hungry implementation for edge computing on portable monitoring devices.

In this work, we propose an artificial-intelligence based SQA method exploiting several SQIs for the identification of the raw abdominal channels carrying the most informative components of the fECG signal in a multi-channel non-invasive abdominal recording, for a lighter fECG extraction and a more reliable fHR estimation. To this aim, this study combined several SQIs and other parameters from the scientific literature and used them as features for a classifier trained to recognize those raw abdominal recordings exhibiting a significant fECG content. The proposed SQI-guided channel selection is aimed at the reduction of the number of channels without *a priori* information on the fetal presentation and orientation and, moreover, it is agnostic with respect to the downstream fECG extraction algorithm. Accordingly, the proposed method could be particularly useful to reduce the computational burden of fECG extraction algorithms in wearable, low-power fetal monitoring devices.

## 2 Materials and methods

A feature extraction step and a feature selection approach for dimensionality reduction were initially carried out on raw multi-channel abdominal recordings to model the proposed supervised SQI-based channel selection approach, as detailed in the Section 2.1. Then, the classifier was selected, trained, and tested on a real dataset of abdominal recordings, as presented in Section 2.2 and Section 2.3. Finally, different figures of merit were introduced in Section 2.4 and used to quantitatively evaluate both the classifier performance and the impact of the proposed channel selection approach on the fetal QRS complex detection.

### 2.1 Feature extraction and selection for the SQI-based channel selection

In order to extract the SQI features and model the SQI-guided channel selection, the following algorithm was conceived. At first, each abdominal signal, sampled at 500 Hz or properly resampled, was segmented to obtain a variable number of 5-s long segments, as in Andreotti et al. (2017). Then, a light preprocessing stage involving a high-pass filtering at 1 Hz by a 4th-order IIR Butterworth filter was applied to suppress eventual low-frequency noises, which is also easy to be managed even in low-power implementations. The signal coming out from this preprocessing contains both the fECG and the mECG, beyond other physiological maternal interferences. For this reason, from the fECG extraction perspective, this preprocessed signal is referred to as “raw” in this work. Remarkably, in this step, we aimed at developing a robust and reliable SQA-based channel selection approach, thus we modelled it by introducing only a high-pass filter with the lowest reasonable cut-off frequency, while allowing the model to deal with powerline interference and all possible high-frequency noises, without limiting the fECG signal band.

Based on the literature on ECG and fECG SQI presented above, several features, both from the time and the frequency domains, as reported in Table 1, were computed over the 5-s preprocessed abdominal segments. In this work, such features were used to train and test a supervised classifier, either considering all of them simultaneously or after a feature selection. Feature selection is a common practice in machine learning to reduce the dimensionality of data by identifying only a subset of optimal features that can effectively model the targeted output. Feature selection may improve or leave the prediction performance unchanged, while allowing for faster and efficient predictors, and a better understanding of the data model (Guyon and Elisseeff, 2003). In this work, a feature selection based on the minimum redundancy maximum relevance (mRMR) algorithm was adopted to rank the features according to their relevance with respect to the response variable (Chandrashekar and Sahin, 2014; Radovic et al., 2017). Specifically, considering a stratified k-fold cross-validation as the one exploited in this work (see Section 2.4), for each data partition, the mRMR relevance score was derived and normalized between 0 and 1. Then, a single relevance score vector was obtained by summing the scores obtained for each feature across all the possible iterations, thus computing a unique relevance value for each feature. Finally, considering all the values in the single relevance score vector, from the highest to the lowest one, the features were ranked according to their relevance contributions and the first *p* features leading to a sufficient amount of the total relevance, from the most to the less important one, were selected.

**TABLE 1:** List of all features extracted from the 5-s long abdominal ECG segments, ranked according to the mRMR-based relevance score.

SQI	Explanation
pband2	average power in the frequency range [10-20] Hz
seSQI	spectral entropy; it is a measure of the spectral power distribution
pband4	average power in the frequency range [48-52] Hz
bas_pow	standard deviation of the baseline extracted using a single-stage moving average filtering on 1-s window
pband3	average power in the frequency range [20-48] Hz
ss	steepest slope in the recording
pband1	average power in the frequency range [0.5-10] Hz
HA	highest amplitude in the recording
stdSQI	standard deviation of the signal; it measures the variation of the amplitude
kSQI	fourth moment of signal, or kurtosis. For a standard, noise-free and normal sinus ECG, the value is less than 5. Low kSQI usually reveals the presence low-frequency noise such as baseline wander, Gaussian noise and power-line interference
pband5	average power in the frequency range [52-100] Hz
LA	lowest amplitude in the recording
pSQI	relative power in the fetal QRS complex, computed as the ratio between the powers in 5–15 Hz and 5–45 Hz bands
complexity	ratio between the mobility of the first derivative of the signals and the mobility of the signal itself, where *mobility* of a given time series x is defined as the square root of the ratio between the variance of the first derivative of x and the variance of x
basSQI	relative power of baseline, computed as the ratio between the powers in 0–3 Hz and 0–100 Hz bands, as in Andreotti et al. (2017)
sSQI	skewness of the signal; it represents the dataset symmetry. If the symmetry is perfect, the skewness is 0. Due to the QRS complexes, ECG is supposed to be highly skewed, whereas low skewness values are expected for the noise, characterized by approximately symmetric distributions. Therefore, skewness is less robust to noise than kurtosis

### 2.2 Classification model

Compared to rule-based approaches, the adoption of machine learning tools allows achieving high robustness by integrating several features to support the decision on the channel selection. In this work, among the possible feature-based supervised classification models, an ensemble tree classifier was adopted for the binary classification related to the SQI-based channel selection task, which is also suitable for low-power device implementations. Specifically, in order to avoid overfitting, default classifier parameters offered in MATLAB were exploited (i.e., the bootstrap aggregation method and 100 ensemble learning cycles, with ten decision splits per tree at maximum), thus possibly providing the readers with a more generic classification model to be used on different datasets. This choice was also made by considering the size of the adopted dataset, which was not large enough to assess any parameters’ optimization procedure.

### 2.3 Dataset for training and testing the SQA-based classification model

In this work, 170 real non-invasive 24-channel abdominal electrophysiological recordings, which were acquired from 55 voluntary pregnant women between the 21st and 27th week of gestation, were used. This gestational epoch was chosen in order to deal with transabdominal recordings potentially leading to the most reliable fECG signals in terms of morphology, with a limited impact of the *vernix caseosa,* which significantly hampers non-invasive fECG extraction from the 28th week of gestation (Oostendorp et al., 1989a; Oostendorp et al., 1989b). The dataset exploited for training and testing the SQA-based classification model was obtained from such signals by extracting 127,992 5-s segments of the raw abdominal channels, successively labelled as described below. Signals were recorded at the Pediatric Cardiology and Congenital Heart Disease Unit of the ARNAS G. Brotzu Hospital in Cagliari (Italy). The study was approved by the Independent Ethics Committee of the Cagliari University Hospital (AOU Cagliari) and performed following the principles outlined in the 1975 Helsinki Declaration, as revised in 2000. All the voluntary pregnant women provided their signed informed consent to the recording, and all the signals came from healthy fetuses.

**FIGURE 1:**
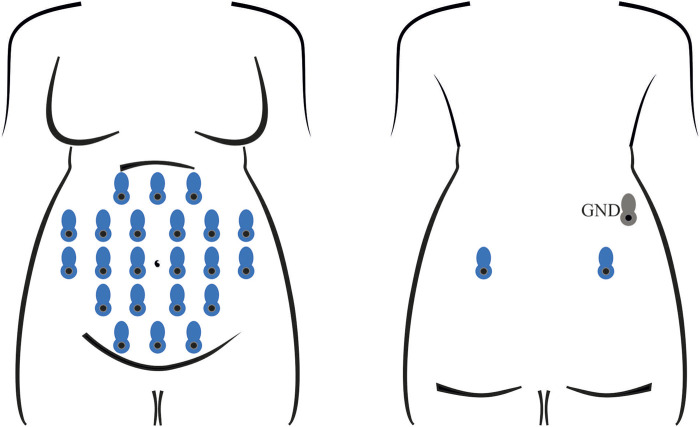
Electrode positioning for the recording of all abdominal signals involved in training and testing the classification model. In each session, 24 unipolar channels were acquired with an average-reference amplifier, exploiting 22 measuring electrodes on the abdomen and two on the back (in blue) of the pregnant volunteer, along with a ground electrode (in grey), also on the back.

Electrophysiological recordings were performed with the Porti7 portable physiological measurement system (TMSi, Netherlands), following the electrode positioning shown in Figure 1. This class IIa medical device, featuring a common average amplifier with DC coupling, simultaneously samples 24 single-ended channels at 2048 Hz, with an input bandwidth limited by the internal digital decimation filter to approximately 550 Hz. The digitization at 22 bits led to a 71.526 nV resolution.

**FIGURE 2:**
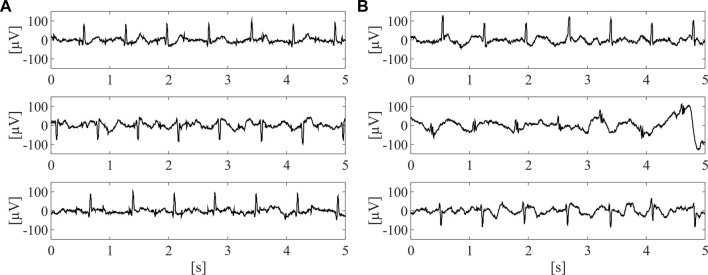
Examples of three informative **(A)** and three non-informative **(B)** 5-s segments included in the dataset adopted for SQA-based classification modelling, after preliminary pre-processing by 4th-order IIR Butterworth high-pass filter at 1 Hz.

On this dataset, a labelling process was carried out in order to train the supervised classifier for the identification of the raw abdominal channels carrying enough fECG information, thus enabling to obtain good quality fECG traces after the application of fully-featured fECG extraction algorithms. Therefore, all labels were defined on fECG signals extracted by the algorithm presented in (Fotiadou et al., 2018). Specifically, fECG extraction was performed by blind source separation, as detailed in (Varanini et al., 2013), then followed by fECG enhancement by time-sequence adaptive filtering, to improve the quality of fECG morphology and SNR (Fotiadou et al., 2018). Hence, an abdominal channel was identified as informative only if the SNR value computed on the extracted fECG signal was above 5 dB. The other channels were labelled as non-informative. Nonetheless, to ensure an accurate training for the classification model, the automatic SNR-based labelling was double-checked by visual inspection and, if necessary, corrected. Because of the prevalence of non-informative labels, a random downsampling process was performed to obtain a balanced dataset composed of 10,336 5-s segments of the raw abdominal channels, equally distributed between informative and non-informative labels. Some examples of informative and non-informative segments included in the dataset are depicted in Figure 2.

### 2.4 Performance evaluation

#### 2.4.1 SQA-based classification performance evaluation

In order to evaluate the SQI-based classifier performance, a 10-time 10-fold cross-validation with stratified partitions was used. Classification results were quantitatively assessed in terms of accuracy (Acc), true positive rate (TPR, or sensitivity), true negative rate (TNR, or specificity), positive predictive value (PPV, or precision), and F1 score. Here, TP and TN denote the number of informative and non-informative channels correctly identified, respectively, false positives (FP) represents non-informative channels erroneously recognized as informative ones, while false negatives (FN) represents informative channels erroneously classified as non-informative ones. Each performance index was evaluated on each fold and time, and the mean and standard deviation values are provided hereafter.

Remarkably, for training and testing the SQI-based classification model, the choice of the 10-time 10-fold cross-validation was aimed at providing the model with a substantial and balanced number of instances in the two classes (informative and non-informative abdominal signals). For a more reliable assessment limiting the bias due to a specific 10-fold subdivision, the 10-fold partition was iterated ten times. Though a leave-one-subject-out could have produced a result with a reduced subject-based bias, the classifier testing was not considered as the only performance index. In fact, in this study, the impact of the SQA-based approach and its eventual weaknesses on different completely unseen subjects were analyzed subsequently, on a different real dataset, in terms of fetal QRS detection performance, thus giving an overview of its potentialities in a real application scenario.

#### 2.4.2 Evaluation of the proposed channel selection approach impact on fHR measurement

To assess the effect of adopting the proposed SQI-based channel selection approach in the typical long-term monitoring scenario, we evaluated its impact over the fetal QRS detection performance. To this aim, we selected a state-of-the-art fetal QRS detector, i.e., the *maxsearch* algorithm available from (Sameni, 2018), in two different conditions:

- on the whole set of abdominal signals,
- only on those signals identified as informative by the proposed SQI-based channel selection.

This test was performed on a set of 50 recordings derived from a publicly available real multi-channel dataset of non-invasive abdominal recordings, i.e., the NInFEA dataset (Sulas et al., 2021), in which the fetal R-peaks were manually annotated. Therefore, after an initial resampling at 512 Hz, all features were extracted channel-wise and provided as input to the classifier.

On this dataset, recorded during a pulsed-wave Doppler simultaneous acquisition, a further pre-processing step was required to deal with a stronger baseline wander. As such, a more aggressive high-pass filtering stage was introduced, which consisted of a 5th-order IIR Butterworth high-pass filter with cut-off frequency of 3 Hz, following (Andreotti et al., 2017). Furthermore, despite some abdominal channels were substantially affected by powerline noise, they were rare cases among the available 24 channels, mainly because of bad contact due to the presence of the active ultrasound probe in the nearby. As such, no notch filtering was introduced also in this case, while preferring to discard these channels by labelling them as non-informative, considering the typical absence of usable fECG in these channels. Then, the fECG component was extracted by means of a multi-reference QRD-RLS adaptive filter, a general-purpose method exhibiting good results in fECG extraction (Baldazzi et al., 2020; Sulas et al., 2020). Here, to extract the fECG component from each abdominal trace, the multi-reference QRD-RLS adaptive filter was fed with three non-coplanar thoracic mECG leads as noise references, which were available for each NInFEA recording, and with the number of taps and the forgetting factor equal to 20 and 0.999, respectively, as in Sulas et al. (2020). The selection of this kind of extraction algorithm allowed us to perform fECG extraction regardless of the number of abdominal channels provided in input, thus without being affected by the number of channels labelled as informative or not. Furthermore, adaptive filters can be simply exploited in portable solutions requiring low computational load.

Fetal R-peaks annotation was performed automatically and then corrected manually, after a preliminary fECG extraction step. Specifically, the fECG extraction and R-peak detection algorithms presented in Jamshidian-Tehrani and Sameni (2018), were exploited, following their implementation released with the NInFEA dataset. Then, for each recording, the detected fetal R-peaks were carefully analyzed by a clinical expert, which visually inspected their annotations in each multichannel-channel trace. For an even more reliable annotation, all fetal R-peaks annotations were further compared to the annotations of the V waves in the synchronous pulsed-wave Doppler acquisition, by considering a clinically reasonable distance between the electrical and the mechanical ventricular activation [i.e., 200 ms, as also in Sulas et al. (2021)]. Indeed, in order to consider abdominal signals lasting at least 11 s and with a trustable fetal R-peaks annotation by simple visual inspection, 50 signals out of 60 were selected and considered for this analysis.

Different figures of merit were computed to evaluate the fetal QRS detection performance obtained on the whole set of abdominal signals and on the informative channels only, as:

$${ACC}_{\det}={TP}_{\det}/\left({TP}_{\det}+{FP}_{\det}+{FN}_{\det}\right)$$

$${TPR}_{\det}={TP}_{\det}/\left({TP}_{\det}+{FN}_{\det}\right)$$

$${PPV}_{\det}={TP}_{\det}/\left({TP}_{\det}+{FP}_{\det}\right)$$

$${F1\;score}_{\det}=\left(2∙{PPV}_{\det}∙{TPR}_{\det}\right)/\left({PPV}_{\det}+{TPR}_{\det}\right)$$

in which ${TP}_{\det}$ denotes the fetal R peaks correctly identified by the detector, ${FN}_{\det}$ the undetected fetal R-peaks and ${FP}_{\det}$ the incorrectly detected ones. For this evaluation, a 50-ms tolerance window was set, by assuming this window length as appropriate to enclose a fetal QRS complex in the gestational age between 20th and 30th weeks (Taylor et al., 2003).

For this latter evaluation, statistical analysis was performed by the non-parametric Kruskal-Wallis test for multiple comparisons and by the Wilcoxon signed rank test for pairwise comparisons. Specifically, in all statistical analyses, a significance level of 5% was considered and the corrected *p*-values were reported according to Bonferroni’s correction.

All data processing and performance analyses were carried out in MATLAB R2022a (MathWorks Inc., MA, United States).

## 3 Results

### 3.1 SQA-based classification results

**FIGURE 3:**
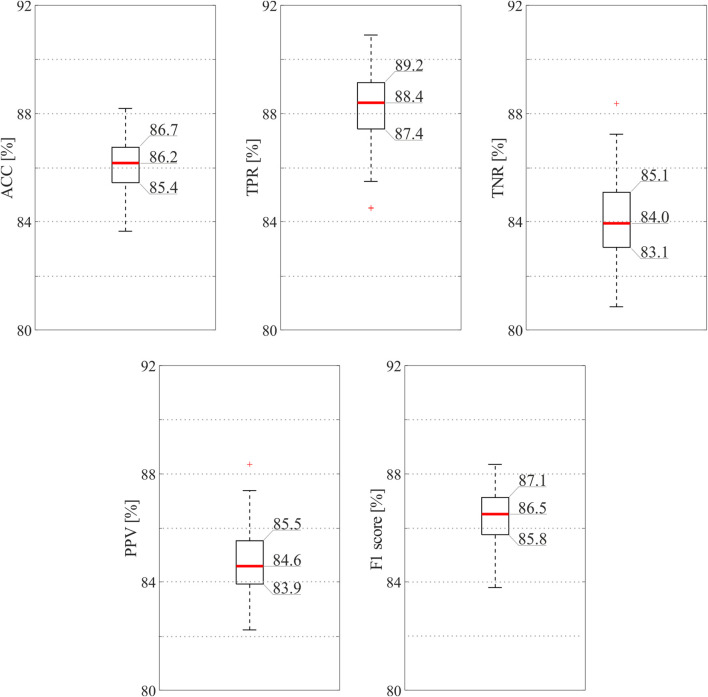
SQI-based classification results obtained by the ensemble tree exploiting all available features. In order to provide the reader with an accurate display of the results, exact values are reported for the 25th and 75th percentiles and for the medians.

Figure 3 reports the results of the classification model when all the 16 features were exploited. As can be seen, the ensemble tree accurately identified informative and non-informative abdominal channels (median ACC = 86.2%) and with high precision (median PPV = 84.6%, median F1 = 86.5%), thus highlighting the robustness of the SQA-based classification approach for the identification of the raw abdominal channels carrying the most informative components of the fECG signal. Interestingly, the proposed model recognized good-quality raw recordings with slightly higher performance than bad-quality ones (median TPR = 88.4%, median TNR = 84.0%).

**FIGURE 4:**
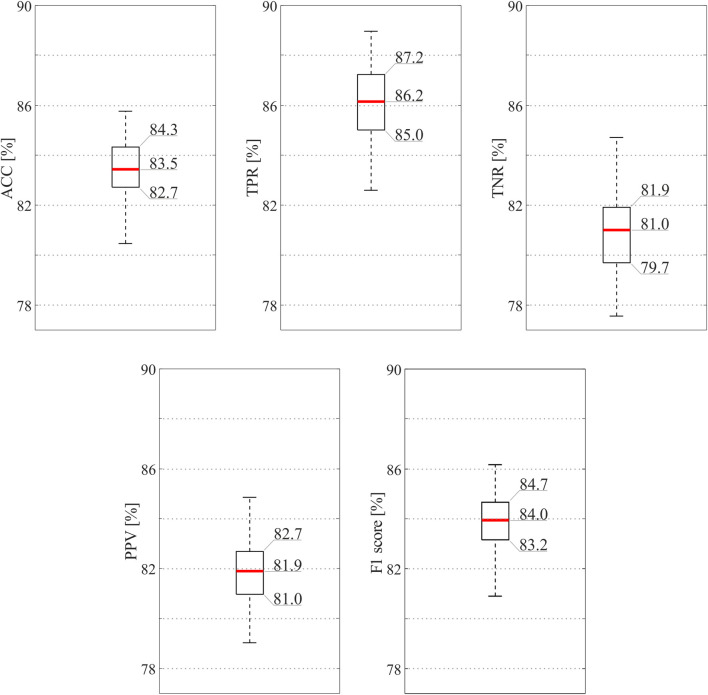
SQI-based classification results obtained by the ensemble tree exploiting only those features selected by the mRMR algorithm. In order to provide the reader with an accurate display of the results, exact values are reported for the 25th and 75th percentiles and for the medians.

Figure 4 depicts the classification performance achieved by the SQI-based model with only the selected features. According to preliminary investigations (data not shown), by considering the 80% of the total relevance, only the features from 1st to 9th in Table 1 were exploited for computing the feature selection findings. As can be seen from Figure 4, results remained high and stable (i.e., median value for ACC = 83.5%, TPR = 86.2%, and TNR = 81.0%), despite the use of a reduced number of features led to slightly lower metrics, with a performance decrease of about 2.6% on average, and less precision overall (median PPV = 81.9%, median F1 = 84.0%). In this case, the most informative channels in terms of fECG contribution could be identified with ACC higher than 83%, so that non-informative channels could be discarded, thus reducing the computational burden for accurate fECG extraction methods downstream, in turn minimizing power consumption in wearable fECG monitors. Nonetheless, the same imbalance between TPR and TNR was preserved, despite the adoption of more features was usually associated to better performance in terms of TNR. Remarkably, even using a subset of features computed on the preprocessed abdominal signals, we were able to achieve good classification results, reducing even more the computational load.

### 3.2 Impact of the proposed SQA-based channel selection approach on fetal QRS detection

**FIGURE 5:**
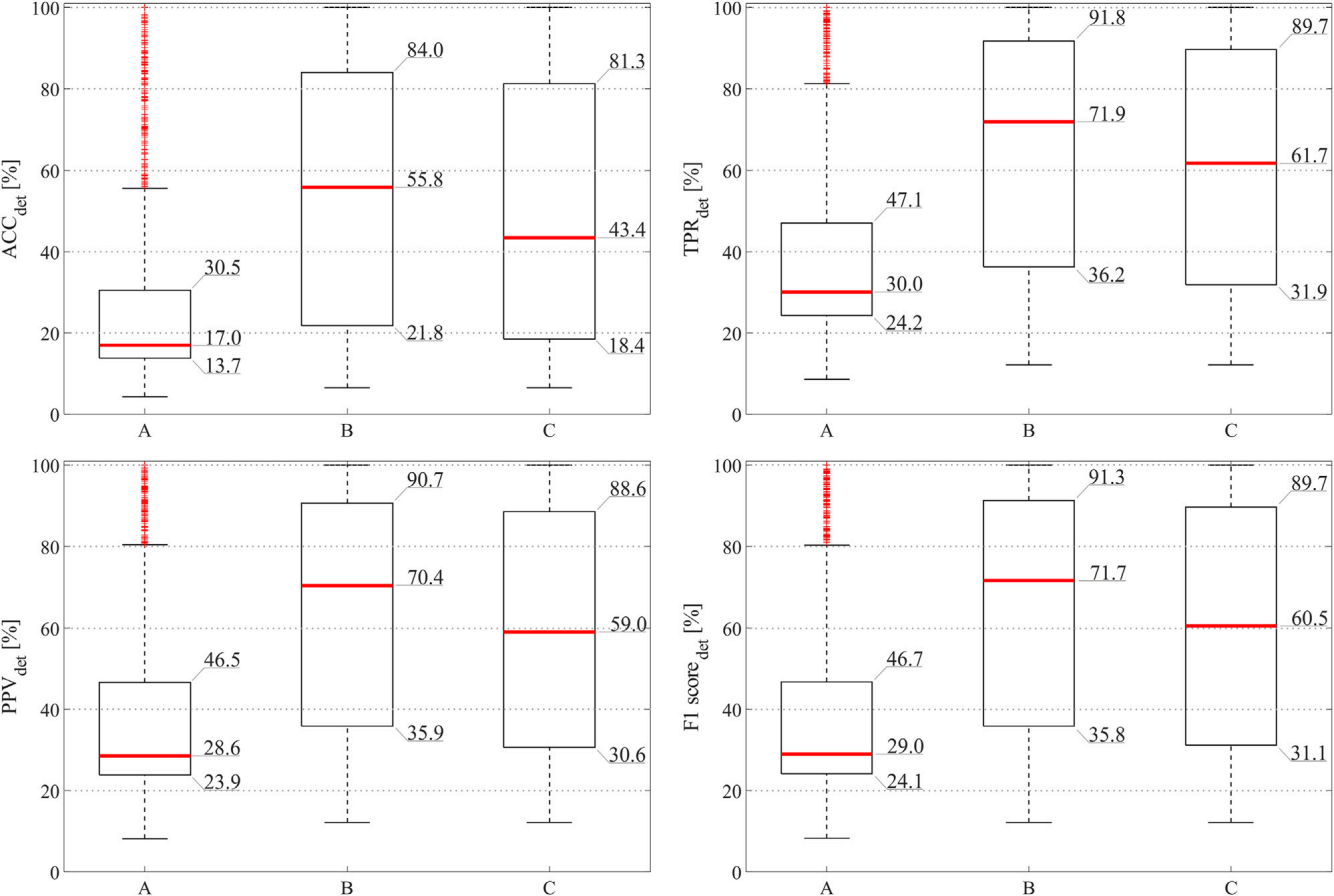
Fetal QRS detection results obtained by considering fECG signals extracted by all available abdominal channels **(A)**, by the abdominal channels identified as informative by the SQA-based approach exploiting all features **(B)**, and by the abdominal channels identified as informative ones by the proposed approach involving selected features only **(C)**.

Figure 5 and Table 2 report the results obtained when assessing the impact of the proposed SQA-based channel selection approach on the NInFEA dataset, in terms of fetal QRS detection performance and number of channels identified as informative, by using either all the available features or those selected by the mRMR approach only.

**TABLE 2:** Number of raw abdominal channels identified as informative by the proposed approach exploiting all features or selected features only, with the clinical information about the week of gestation and the fetal presentation of the recordings (L: left, R: right, O: occiput, S: sacrum, T: transverse, P: posterior A: anterior). Data information for the adopted 50 recordings was taken from Sulas et al. (2021). Among the signals, some of them were not used because of unreliable fetal QRS annotation (marked with *) or because of too short duration (marked with^‡^).

Signals # in NInFEA	Subject	Gestational week	Fetal presentation	Number of channels identified as informative (approach using all features)	Number of channels identified as informative (approach using selected features)
1	1	27	vertex, OT	1	0
2				0	2
3				4	5
4	2	25	vertex, ROT	0	0
5				0	0
6	3	21 + 1	vertex, ROT	2	2
7				0	0
8	4	22 + 4	vertex, LOT	7	6
9^∗^	5	24	vertex, LOT	not used	not used
10	6	24 + 4	breech, LST	5	3
11				4	8
12^∗^	7	25 + 4	breech, LSA	not used	not used
13	8	21 + 5	breech, RSP	7	9
14				8	6
15	9	25 + 2	vertex, ROP	8	7
16				7	7
17	10	24	vertex, ROP	0	0
18^∗^	11	26 + 6	breech, RST	not used	not used
19	12	22 + 4	breech, LSP	3	4
20	13	26 + 2	breech, LST	4	8
21				2	9
22^∗^	14	25 + 1	vertex, LOP	not used	not used
23	15	24 + 1	breech, RSP	2	3
24	16	26 + 6	breech, LSP	5	5
25	17	26 + 3	vertex, LOP	1	2
26	18	24 + 1	vertex, LOP	5	4
27^∗^	19	27 + 1	vertex, LOP	not used	not used
28	20	26 + 6	vertex, ROP	8	9
29				8	9
30	21	22 + 3	breech, LSP	6	6
31	22	27 + 5	vertex, ROP	4	7
32				4	7
33				4	7
34	23	25	breech, RSP	0	0
35	24	27	vertex, ROP	2	3
36				2	4
37	25	25 + 1	vertex, LOP	1	2
38^‡^				not used	not used
39	26	24	vertex, OA	4	5
40	27	21 + 3	breech, RSP	8	10
41				9	10
42^‡^	28	24 + 6	breech, LSP	not used	not used
43	29	23 + 4	vertex, ROP	7	8
44				6	8
45	30	23	vertex, OP	6	3
46	31	24 + 4	breech, LSP	11	11
47	32	21 + 1	vertex, LP	12	10
48				12	12
49^‡^	33	22	breech, LSP	not used	not used
50				4	9
51	34	24 + 2	vertex, LOP	9	7
52				10	8
53	35	21	vertex, LOP	7	6
54				5	7
55	36	25	breech, LSP	8	9
56				8	10
57	37	23 + 6	vertex, OA	7	8
58	38	23	vertex, ROP	13	13
59^∗^	39	27 + 3	vertex, LOP	not used	not used
60^∗^				not used	not used

**FIGURE 6:**
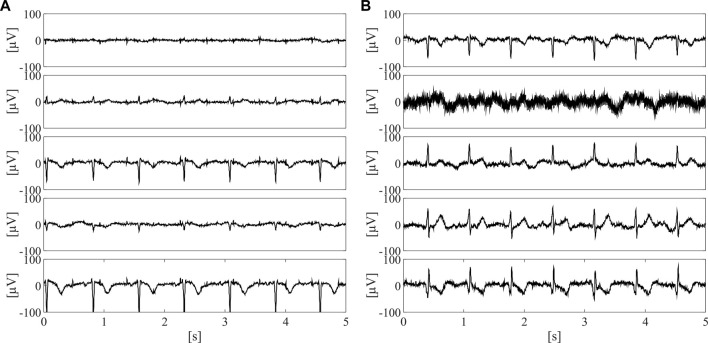
Five 5-s long abdominal segments from the subjects showing the highest [i.e., the 38th, **(A)**] and the lowest number of informative signals [i.e., the 2^nd^, **(B)**] after preprocessing.

As can be seen from Figure 5, the adoption of the proposed approach significantly improved the fetal QRS detection performance with respect to considering all available raw abdominal channels (*p* < 0.0001 for all metrics), leading to an average improvement across all metrics of 41.3% when all features are maintained, and of 30.0% when only the selected ones are considered. However, no statistical significance was found when comparing the use of all available features and only those selected by mRMR-based approach. Conversely, when looking at Table 2, it is evident that the number of selected abdominal channels by the proposed approach when considering all features or only those selected by the mRMR-based approach is quite coherent, but independent from the gestational age and fetal presentation, with 5 ± 3 channels (mean ± standard deviation) selected when all features are considered, and 6 ± 3 when only the selected ones were retained across the 50 examined multi-channel recordings. For the sake of the completeness, some abdominal segments from the subjects showing the highest and the lowest number of signals identified as informative by our approach (i.e., the 38th and the 2nd in Table 2, respectively) are depicted in Figure 6.

## 4 Discussion

In this work, several time-domain and frequency-domain SQIs from the scientific literature were exploited to train a supervised machine-learning approach for the selection of the raw, non-invasive abdominal channels carrying the significant fetal contributions. To the best of the authors’ knowledge, this represent the first study looking at the quality of the abdominal channels by means of machine learning approaches to select the best ones to be used for subsequent fECG extraction. Although feature extraction and classification were performed offline in this study, they can be also implemented in real-time on digital signal processing architectures, according to the literature in different biomedical engineering fields (Pani et al., 2016).

From our results, the SQA-based classification approach revealed high ACC, above 86% when all features were considered, correctly identifying more than 88% of the informative abdominal signals (see Figure 3). The proposed method seemed to be more conservative and high-performing when all features were considered, but good identification performance was obtained also when only a restricted number of features were taken into account (see Figure 4). Nonetheless, despite our findings seemed to be less accurate than previous works (Mertes et al., 2022) where 94% of ACC, PPV, and TPR were found, it should be noted that a different dataset with a lower number of abdominal traces was used to model a CNN-based approach, which conversely could be hardly exploited in wearable, low-power fECG monitoring devices. Indeed, our approach is aimed at limiting data complexity by reducing the number of abdominal channels to be processed for fECG extraction to those effectively carrying information on the fECG signal. Remarkably, this SQA-based data reduction could be extended even more, by stopping the processing when no reliable, good channels are identified in input, by following the idea presented in Orphanidou et al. (2015).

Furthermore, by looking at the possible impact of our method on fetal QRS detection, it is evident that the proposed approach introduced a significant improvement in all evaluated metrics, even outperforming previous scientific literature in this regard. Specifically, in Liu et al. (2014), the authors developed a multi-step method based on SQA to provide accurate maternal and fetal QRS complexes locations from abdominal recordings. However, the adoption of a SQA approach for the abdominal channel selection, to be later processed, introduced an improvement slightly above 5% in F1 score for fetal QRS detection. Despite the use of a different dataset [i.e., the PhysioNet/Computing in Cardiology Challenge 2013 (Goldberger et al., 2000; Silva et al., 2013; Clifford et al., 2014)], and a more elaborated fECG extraction algorithm, the F1 score increase was definitely lower than this work. In fact, in this work, fECG extraction was performed by the multi-reference QRD-RLS adaptive filter as set in Sulas et al. (2020), which is not expressly conceived for fECG extraction, although able to provide excellent results.

## 5 Conclusion

In this work, a machine learning approach for the SQA of non-invasive, multi-channel abdominal recordings was presented, aiming at driving the channel selection to feed fully featured fECG extraction algorithms. This aspect, which was proven to significantly enhance fetal QRS detection performance, also plays a key role in reducing the power consumption associated with data processing in real-time fetal ECG monitors, paving the way to the development of efficient, wearable, low-power devices for fHR surveillance. Additionally, the proposed approach can be used to identify the most-informative channels in high-density recordings, or the best electrode positioning from repeated measurements with a low number of channels. This is particularly relevant when the recordings are blinded to the fetal presentation, and could allow dealing with substantial changes in the fetal presentation, especially affecting the recordings in early pregnancy.

## Data Availability

Publicly available datasets were analyzed in this study. This data can be found here: https://www.physionet.org/content/ninfea/1.0.0/.

## References

[B1] Agostinelli A. Grillo M. Biagini A. Giuliani C. Burattini L. Fioretti S. (2015). Noninvasive fetal electrocardiography: An overview of the signal electrophysiological meaning, recording procedures, and processing techniques. Ann. noninvasive Electrocardiol. Off. J. Int. Soc. Holter Noninvasive Electrocardiol. Inc. 20 (4), 303–313. 10.1111/anec.12259 PMC6931562 25640061

[B2] Allen J. Murray A. (1996). Assessing ECG signal quality on a coronary care unit. Physiol. Meas. 17 (4), 249–258. 10.1088/0967-3334/17/4/002 8953623

[B3] Andreotti F. Graser F. Malberg H. Zaunseder S. (2017). Non-invasive fetal ECG signal quality assessment for multichannel heart rate estimation. IEEE Trans. Biomed. Eng. 64 (12), 2793–2802. 10.1109/tbme.2017.2675543 28362581

[B4] Baldazzi G. Sulas E. Urru M. Tumbarello R. Raffo L. Pani D. (2020). Wavelet denoising as a post-processing enhancement method for non-invasive foetal electrocardiography. Comput. Methods Programs Biomed. 195, 105558. 10.1016/j.cmpb.2020.105558 32505973

[B5] Behar J. Oster J. Li Q. Clifford G. D. (2012). A single channel ECG quality metric. Computing in Cardiology, 381–384.

[B6] Behar J. Oster J. Li Q. Clifford G. D. (2013). ECG signal quality during arrhythmia and its application to false alarm reduction. IEEE Trans. Biomed. Eng. 60 (6), 1660–1666. 10.1109/tbme.2013.2240452 23335659

[B7] Behar J. A. Weiner Z. Warrick P. (2019) “Special session on computational fetal monitoring,” in 2019 Computing in Cardiology (CinC), Singapore. 1–4. 10.22489/CinC.2019.030

[B8] Chandrashekar G. Sahin F. (2014). A survey on feature selection methods. Comput. Electr. Eng. 40 (1), 16–28. 10.1016/j.compeleceng.2013.11.024

[B9] Clifford G. D. Behar J. Li Q. Rezek I. (2012). Signal quality indices and data fusion for determining clinical acceptability of electrocardiograms. Physiol. Meas. 33 (9), 1419–1433. 10.1088/0967-3334/33/9/1419 22902749

[B10] Clifford G. D. Silva I. Behar J. Moody G. B. (2014). Non-invasive fetal ECG analysis. Physiol. Meas. 35 (8), 1521–1536. 10.1088/0967-3334/35/8/1521 25071093 PMC4164169

[B11] Daluwatte C. Johannesen L. Galeotti L. Vicente J. Strauss D. G. Scully C. G. (2016). Assessing ECG signal quality indices to discriminate ECGs with artefacts from pathologically different arrhythmic ECGs. Physiol. Meas. 37 (8), 1370–1382. 10.1088/0967-3334/37/8/1370 27454007 PMC4975422

[B12] Del Rio B. A. S. Lopetegi T. Romero I. (2011). Assessment of different methods to estimate electrocardiogram signal quality. Computing in Cardiology. IEEE, 609–612.

[B13] Di Marco L. Y. Duan W. Bojarnejad M. Zheng D. King S. Murray A. (2012). Evaluation of an algorithm based on single-condition decision rules for binary classification of 12-lead ambulatory ECG recording quality. Physiol. Meas. 33 (9), 1435–1448. 10.1088/0967-3334/33/9/1435 22902810

[B14] Donofrio M. T. Moon-Grady A. J. Hornberger L. K. Copel J. A. Sklansky M. S. Abuhamad A. (2014). Diagnosis and treatment of fetal cardiac disease. Circulation 129 (21), 2183–2242. 10.1161/01.cir.0000437597.44550.5d 24763516

[B15] Fotiadou E. van Laar J. O. E. H. Oei S. G. Vullings R. (2018). Enhancement of low-quality fetal electrocardiogram based on time-sequenced adaptive filtering. Med. Biol. Eng. Comput. 56 (12), 2313–2323. 10.1007/s11517-018-1862-8 29938302 PMC6245004

[B16] Fotiadou E. van Sloun R. J. G. van Laar J. O. E. H. Vullings R. (2021). A dilated inception CNN-LSTM network for fetal heart rate estimation. Physiol. Meas. 42 (4), 045007. 10.1088/1361-6579/abf7db 33853039

[B17] Gaikwad R. Warren J. R. (2009). The role of home-based information and communications technology interventions in chronic disease management: A systematic literature review. Health Inf. J. 15, 122–146. 10.1177/1460458209102973 19474225

[B18] Goldberger A. L. Amaral L. A. N. Glass L. Hausdorff J. M. Ivanov P. C. Mark R. G. (2000). PhysioBank, PhysioToolkit, and PhysioNet: Components of a new research resource for complex physiologic signals. Circulation 101 (23), e215–e220. 10.1161/01.cir.101.23.e215 10851218

[B19] Guyon I. Elisseeff A. (2003). An introduction to variable and feature selection. J. Mach. Learn Res., 1157–1182.

[B20] Hayn D. Jammerbund B. Schreier G. (2012). QRS detection based ECG quality assessment. Physiol. Meas. 33 (9), 1449–1461. 10.1088/0967-3334/33/9/1449 22902864

[B21] Jagannath D. J. Selvakumar A. I. (2014). Issues and research on foetal electrocardiogram signal elicitation. Biomed. Signal Process Control 10, 224–244. 10.1016/j.bspc.2013.11.001

[B22] Jamshidian-Tehrani F. Sameni R. (2018). Fetal ECG extraction from time-varying and low-rank noninvasive maternal abdominal recordings. Physiol. Meas. 39 (12), 125008. 10.1088/1361-6579/aaef5d 30523836

[B23] Jaros R. Martinek R. Kahankova R. (2018). Non-adaptive methods for fetal ECG signal processing: A review and appraisal. Sensors 18 (11), 3648. 10.3390/s18113648 30373259 PMC6263968

[B24] Johannesen L. Galeotti L. (2012). Automatic ECG quality scoring methodology: Mimicking human annotators. Physiol. Meas. 33 (9), 1479–1489. 10.1088/0967-3334/33/9/1479 22902927

[B25] Johnson A. E. W. Behar J. Andreotti F. Clifford G. D. Oster J. (2015). Multimodal heart beat detection using signal quality indices. Physiol. Meas. 36 (8), 1665–1677. 10.1088/0967-3334/36/8/1665 26218060

[B26] Kahankova R. Martinek R. Jaros R. Behbehani K. Matonia A. Jezewski M. (2020). A review of signal processing techniques for non-invasive fetal electrocardiography. IEEE Rev. Biomed. Eng. 13, 51–73. 10.1109/rbme.2019.2938061 31478873

[B27] Karimi Rahmati A. Setarehdan S. K. Araabi B. N. (2017). A PCA/ICA based fetal ECG extraction from mother abdominal recordings by means of a novel data-driven approach to fetal ECG quality assessment. J. Biomed. Phys. Eng. 7 (1), 37–50. 28451578 PMC5401132

[B28] Langley P. Di Marco L. Y. King S. Duncan D. Di Maria C. Duan W. (2011). An algorithm for assessment of quality of ECGs acquired via mobile telephones. Computing in Cardiology. IEEE, 281–284.

[B29] Lanzola G. Ginardi M. G. Mazzanti A. Quaglini S. (2014). Gquest: Modeling patient questionnaires and administering them through a mobile platform application. Comput. Methods Programs Biomed. 117 (2), 277–291. 10.1016/j.cmpb.2014.07.010 25154645

[B30] Li Q. Clifford G. D. (2012). Signal quality and data fusion for false alarm reduction in the intensive care unit. J. Electrocardiol. 45 (6), 596–603. 10.1016/j.jelectrocard.2012.07.015 22960167

[B31] Li Q. Mark R. G. Clifford G. D. (2007). Robust heart rate estimation from multiple asynchronous noisy sources using signal quality indices and a Kalman filter. Physiol. Meas. 29 (1), 15–32. 10.1088/0967-3334/29/1/002 18175857 PMC2259026

[B32] Li Q. Rajagopalan C. Clifford G. D. (2014). A machine learning approach to multi-level ECG signal quality classification. Comput. Methods Programs Biomed. 117 (3), 435–447. 10.1016/j.cmpb.2014.09.002 25306242

[B33] Liu C. Li P. Di Maria C. Zhao L. Zhang H. Chen Z. (2014). A multi-step method with signal quality assessment and fine-tuning procedure to locate maternal and fetal QRS complexes from abdominal ECG recordings. Physiol. Meas. 35 (8), 1665–1683. 10.1088/0967-3334/35/8/1665 25069817

[B34] Liu C. Zhang X. Zhao L. Liu F. Chen X. Yao Y. (2018). Signal quality assessment and lightweight QRS detection for wearable ECG SmartVest system. IEEE Internet Things J. 6 (2), 1363–1374. 10.1109/jiot.2018.2844090

[B35] Majumder S. Mondal T. Deen M. J. (2017). Wearable sensors for remote health monitoring. Sensors (Basel). 17 (1), 130. 10.3390/s17010130 28085085 PMC5298703

[B36] Martinez-Tabares F. J. Espinosa-Oviedo J. Castellanos-Dominguez G. (2012). “Improvement of ECG signal quality measurement using correlation and diversity-based approaches,” in Annual International Conference of the IEEE Engineering in Medicine and Biology Society (IEEE), 4295–4298. 10.1109/EMBC.2012.6346916 23366877

[B37] Mertes G. Long Y. Liu Z. Li Y. Yang Y. Clifton D. A. (2022). A deep learning approach for the assessment of signal quality of non-invasive foetal electrocardiography. Sensors (Basel). 22 (9), 3303. 10.3390/s22093303 35591004 PMC9103336

[B38] Mhajna M. Schwartz N. Levit-Rosen L. Warsof S. Lipschuetz M. Jakobs M. (2020). Wireless, remote solution for home fetal and maternal heart rate monitoring. Am. J. Obstet. Gynecol. MFM 2 (2), 100101. 10.1016/j.ajogmf.2020.100101 33345967

[B39] Moody B. E. (2011). “Rule-based methods for ECG quality control,” in 2011 computing in Cardiology (IEEE), 361–363.

[B40] Oostendorp T. F. van Oosterom A. Jongsma H. W. (1989). Electrical properties of tissues involved in the conduction of foetal ECG. Med. Biol. Eng. Comput. 27 (3), 322–324. 10.1007/bf02441492 2601455

[B41] Oostendorp T. F. Van Oosterom A. Jongsma H. W. (1989). The effect of changes in the conductive medium on the fetal ECG throughout gestation. Clin. Phys. Physiol. Meas. 10, 11–20. 10.1088/0143-0815/10/4b/002 2630157

[B42] Orphanidou C. Bonnici T. Charlton P. Clifton D. Vallance D. Tarassenko L. (2015). Signal-quality indices for the electrocardiogram and photoplethysmogram: Derivation and applications to wireless monitoring. IEEE J. Biomed. Heal Inf. 19 (3), 832–838. 10.1109/JBHI.2014.2338351 25069129

[B43] Pani D. Barabino G. Citi L. Meloni P. Raspopovic S. Micera S. (2016). Real-time neural signals decoding onto off-the-shelf DSP processors for neuroprosthetic applications. IEEE Trans. Neural Syst. Rehabil. Eng. 24, 993–1002. 10.1109/tnsre.2016.2527696 27164593

[B44] Pani D. Barabino G. Raffo L. (2013). NInFEA: An embedded framework for the real-time evaluation of fetal ECG extraction algorithms. Biomed. Technik/Biomedical Eng. 58, 13. 10.1515/bmt-2012-0018 23314497

[B45] Peters M. Crowe J. Pieri J. F. Quartero H. Hayes-Gill B. James D. (2001). Monitoring the fetal heart non-invasively: A review of methods. J. Perinat. Med. 29 (5), 408–416. 10.1515/jpm.2001.057 11723842

[B46] Radovic M. Ghalwash M. Filipovic N. Obradovic Z. (2017). Minimum redundancy maximum relevance feature selection approach for temporal gene expression data. BMC Bioinforma. 18 (1), 9–14. 10.1186/s12859-016-1423-9 PMC5209828 28049413

[B47] Redmond S. J. Lovell N. H. Basilakis J. Celler B. G. (2008). ECG quality measures in telecare monitoring. Annu. Int. Conf. IEEE Eng. Med. Biol. Soc. IEEE Eng. Med. Biol. Soc. Annu. Int. Conf. 2008, 2869–2872. 10.1109/IEMBS.2008.4649801 19163304

[B48] Rooijakkers M. (2020). “Innovative devices and techniques for multimodal fetal health monitoring,” in Innovative technologies and signal processing in perinatal medicine. Editor Springer. Volume 1. 1st ed, 147.

[B49] Sameni R. Clifford G. D. (2010). A review of fetal ECG signal processing; issues and promising directions. Open Pacing Electrophysiol. Ther. J. 3, 4–20. 10.2174/1876536X01003010004 21614148 PMC3100207

[B50] Sameni R. (2018). The open-source electrophysiological toolbox (OSET). version 3.14 [Internet]Available from: https://gitlab.com/rsameni/OSET/.

[B51] Satija U. Ramkumar B. Manikandan M. S. (2018). A review of signal processing techniques for electrocardiogram signal quality assessment. IEEE Rev. Biomed. Eng. 11, 36–52. 10.1109/rbme.2018.2810957 29994590

[B52] Shahriari Y. Fidler R. Pelter M. M. Bai Y. Villaroman A. Hu X. (2018). Electrocardiogram signal quality assessment based on structural image similarity metric. IEEE Trans. Biomed. Eng. 65 (4), 745–753. 10.1109/tbme.2017.2717876 28644794 PMC5912885

[B53] Shi X. Yamamoto K. Ohtsuki T. Matsui Y. Owada K. (2022). Non-invasive fetal ECG signal quality assessment based on unsupervised learning approach. Annu. Int. Conf. IEEE Eng. Med. Biol. Soc. IEEE Eng. Med. Biol. Soc. Annu. Int. Conf. 2022, 1296–1299. 10.1109/EMBC48229.2022.9870908 36086629

[B54] Silva I. Behar J. Sameni R. Zhu T. Oster J. Clifford G. D. (2013). “Noninvasive fetal ECG: The PhysioNet/computing in cardiology Challenge,” in 2013 computing in Cardiology (CInC) (United States, 149–152. PMC4230703 25401167

[B55] Silva I. Moody G. B. Celi L. (2011). “Improving the quality of ECGs collected using mobile phones: The Physionet/Computing in Cardiology Challenge 2011,” in 2011 computing in Cardiology (IEEE), 273–276.

[B56] Sulas E. Urru M. Tumbarello R. Raffo L. Pani D. (2020). Systematic analysis of single- and multi-reference adaptive filters for non-invasive fetal electrocardiography. Math. Biosci. Eng. 17 (1), 286–308. 10.3934/mbe.2020016 31731352

[B57] Sulas E. Urru M. Tumbarello R. Raffo L. Sameni R. Pani D. (2021). A non-invasive multimodal foetal ECG–Doppler dataset for antenatal cardiology research. Sci. Data 8 (1), 30. 10.1038/s41597-021-00811-3 33500414 PMC7838287

[B58] Taylor M. J. O. Smith M. J. Thomas M. Green A. R. Cheng F. Oseku-Afful S. (2003). Non-invasive fetal electrocardiography in singleton and multiple pregnancies. BJOG 110 (7), 668–678. 10.1046/j.1471-0528.2003.02005.x 12842058

[B59] Varanini M. Tartarisco G. Balocchi R. Macerata A. Pioggia G. Billeci L. (2017). A new method for QRS complex detection in multichannel ECG: Application to self-monitoring of fetal health. Comput. Biol. Med. 85, 125–134. 10.1016/j.compbiomed.2016.04.008 27106501

[B60] Varanini M. Tartarisco G. Billeci L. Macerata A. Pioggia G. Balocchi R. (2013). A multi-step approach for non-invasive fetal ECG analysis. Comput. Cardiol., 281–284.

[B61] Wang J. Y. (2002). “A new method for evaluating ECG signal quality for multi-lead arrhythmia analysis,” in Computers in Cardiology (IEEE), 85–88.

[B62] Zhao Z. Zhang Y. (2018). SQI quality evaluation mechanism of single-lead ECG signal based on simple heuristic fusion and fuzzy comprehensive evaluation. Front. Physiol. 9, 727. 10.3389/fphys.2018.00727 29962962 PMC6011094

